# Leveraging RNA interference technology for selective and sustainable crop protection

**DOI:** 10.3389/fpls.2024.1502015

**Published:** 2024-12-24

**Authors:** Hong-Yue Qi, Dan-Dan Zhang, Binhui Liu, Jie-Yin Chen, Dongfei Han, Dan Wang

**Affiliations:** ^1^ The State Key Laboratory for Biology of Plant Diseases and Insect Pests, Institute of Plant Protection, Chinese Academy of Agricultural Sciences, Beijing, China; ^2^ Western Agricultural Research Center, Chinese Academy of Agricultural Sciences, Changji, China; ^3^ Key Laboratory of Crop Drought Resistance Research of Hebei Province/Institute of Dryland Farming, Hebei Academy of Agriculture and Forestry Sciences, Hengshui, China; ^4^ School of Environmental Science and Engineering, Suzhou University of Science and Technology, Suzhou, China; ^5^ State Key Laboratory of Subtropical Silviculture, School of Forestry and Biotechnology, Zhejiang A & F University, Hangzhou, China

**Keywords:** small interfering RNA, microRNA, gene silencing, crop protection, RNA interference

## Abstract

Double-stranded RNA (dsRNA) has emerged as key player in gene silencing for the past two decades. Tailor-made dsRNA is now recognized a versatile raw material, suitable for a wide range of applications in biopesticide formulations, including insect control to pesticide resistance management. The mechanism of RNA interference (RNAi) acts at the messenger RNA (mRNA) level, utilizing a sequence-dependent approach that makes it unique in term of effectiveness and specificity compared to conventional agrochemicals. Two primary categories of small RNAs, known as short interfering RNAs (siRNAs) and microRNAs (miRNAs), function in both somatic and germline lineages in a broad range of eukaryotic species to regulate endogenous genes and to defend the genome from invasive nucleic acids. Furthermore, the application of RNAi in crop protection can be achieved by employing plant-incorporated protectants through plant transformation, but also by non-transformative strategies such as the use of formulations of sprayable RNAs as direct control agents, resistance factor repressors or developmental disruptors. This review explores the agricultural applications of RNAi, delving into its successes in pest-insect control and considering its broader potential for managing plant pathogens, nematodes, and pests. Additionally, the use of RNAi as a tool for addressing pesticide-resistant weeds and insects is reviewed, along with an evaluation of production costs and environmental implications.

## 1 Introduction

The phenomenon of RNA-induced gene silencing (RNAi) gained prominence following its disclosed mechanism in pests, but it was in tobacco (*Nicotiana tabacum*) plants that this phenomenon was initially documented and published as early as 1928 (Wingard, 1928). This groundbreaking discovery has revolutionized our understanding of gene regulation and its implications for various biological processes, particularly gene silencing via RNAi. Harnessing this process has shown promise in diverse applications, including gene knock down, disease treatment, and crop improvement. Over the last decade, substantial efforts have been made to exploit RNAi for the development of novel crop protection methods. Moreover, RNAi technology has facilitated the creation of genetically modified crops with enhanced traits such as increased yield, improved nutritional content, and prolonged shelf life (Kumar et al., 2020).

Sustainable agriculture entails the development and implementation of environmentally friendly technologies and practices that are readily accessible and advantageous to farmers in terms of crop improvement and productivity (Fletcher et al., 2020). To minimize the adverse effects of synthetic pesticides on health and the environment, there has been a shift towards employing bio-based alternatives. This shift aims to promote agricultural sustainability, leading to the adoption of more environmentally friendly and innovative crop protection strategies by the scientific community. Plant genetic engineering has emerged as a promising avenue to address food shortage and mitigate the impact of plant stresses. Notably, the development of transgenic crops employing advanced biotechnological techniques, including RNAi, has been a significant contribution in this regard. Transgenic RNAi crops are still regarded as GM crops. Provided that Double-stranded RNA (dsRNA)-based products contain no GM organisms (e.g., bacteria for dsRNA production), they are not regarded as genetically modified organisms (GMOs). Interestingly, recent developments using exogenous dsRNA spray to control pathogens and pests have provided a non-transgenic alternative to GMOs (Rajam, 2020; Rodrigues and Petrick, 2020). Furthermore, RNAi induces the silencing of target genes, which is more advantageous than genome editing tools (Arpaia et al., 2020). These distinctive features of RNAi have made it a popular and effective strategy for crop enhancement and protection (Arpaia et al., 2020; Rajam, 2020).

In this review, we explained the mechanism of RNAi-mediated gene silencing and furnished a comprehensive report of the role of RNAi in protecting crops against diverse biotic stresses. Furthermore, we have directed attention towards the potential of RNAi as an innovative and potent alternative for global crop protection strategy, as well as its application in the development of futuristic smart crops resilient to various biotic stresses.

## 2 The functional basis of RNA interference

The ability of RNAi to specifically inhibit gene expression arises from the design 21-25 bp dsRNAs, ensuring only the intended target gene is silenced. The specificity grants RNAi a wide range of potential applications for genetic studies and agriculture, including the protection of beneficial insects against viruses and parasites (San-Miguel and Scott, 2016; Mehlhorn et al., 2021), novel and highly specific insect and parasites control, and traditional plant-incorporated protectants (PIPs: i.e. transgenic plants/RNAi-based plant traits) (Walawage et al., 2013). Another crucial aspect of RNAi is its conservation across different species, indicating its versatile use in manipulating gene expression not only within an organism but also for the organism’s survival and adaptation.

**Figure 1:**
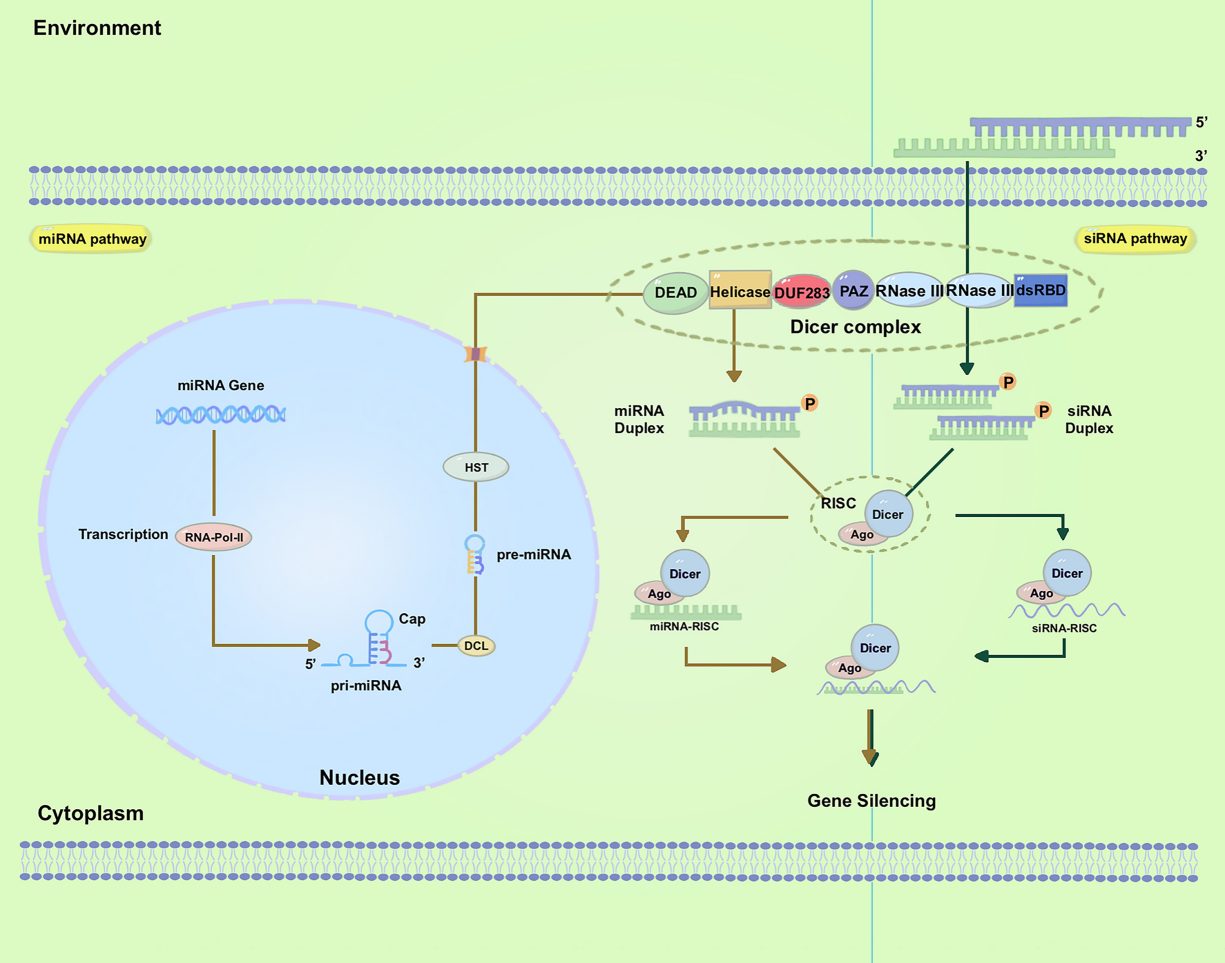
Biogenesis of small RNAs and mechanism of gene silencing. The left panel demonstrates miRNA biogenesis and gene silencing. The right panel shows siRNA biogenesis and gene silencing; Abbreviations used in the figures: dsRNA, double-stranded RNA; siRNA, small-interfering RNA; RISC, RNAi-induced silencing complex; mRNA, messenger RNA; AGO, Argonaute; RNA Pol II, RNA polymerase II; PAZ, PIWI/ARGONAUTE/ZWILLE; DUF283, domain of unknown function 283; dsRBD, dsRNA-binding domain; pre-miRNAs, precursor microRNAs; HST, HASTY.

RNAi regulates gene expression through small noncoding RNAs (sRNAs) (Rajam, 2020) (**Figure 1**). These sRNAs identify the target messenger RNA (mRNA) via homology-based binding and facilitate its degradation with effector proteins. Within the RNAi pathway, there are two primary classes of sRNAs: short-interfering RNAs (siRNAs) and micro-RNAs (miRNAs) (Margis et al., 2006; Borges and Martienssen, 2015; Mamta and Rajam, 2018). While the basic pathway for sRNA biogenesis involves trimming long dsRNAs to form sRNAs, the type of sRNA present is determined by the source of the dsRNA (Parker and Barford, 2006). The application of RNA-based products for insect management requires dsRNA longer than 50 bp but not sRNAs (Ivashuta et al., 2015; Yoshida et al., 2023; Chi et al., 2023), although some studies have shown that sRNA can trigger gene silencing (Gong et al., 2013; Liu S. et al., 2021). In contrast, fungi and plants take up both dsRNAs and sRNAs (Wang and Jin, 2017; Subha et al., 2023; McLaughlin et al., 2023), suggesting different uptake mechanisms for these organisms (Wang and Jin, 2017).

siRNAs were originally observed during transgene- and virus-induced silencing in plants (Mello and Conte, 2004). They are generally formed from the dsRNA obtained from various sources, including viruses, transposons, transgenes, aberrant mRNAs, and inverted repeats (IRs), among others (Preall and Sontheimer, 2005; Matranga and Zamore, 2007; Golden et al., 2008; Wilson and Doudna, 2013). In addition to siRNAs, a multitude of miRNAs have been identified in various eukaryotes, and their sequences are available online. miRNAs are short, endogenous, single-strand RNAs, typically 21-24 nucleotides long, derived from hairpin transcripts. They play a regulatory role in gene expression in animals and plants (Bartel, 2009; Feng and Riddle, 2020). Furthermore, multiple miRNAs can regulate the same gene (Samad et al., 2021). The miRNA pathway operates at the post-transcriptional level and is involved in a range of physiological and pathophysiological processes (Ambros, 2004; Yu et al., 2017). Even though siRNAs and miRNAs were first found in separate research studies, these two small RNAs are closely connected in their biological functions, formation of RNA-protein complexes, and capacity to regulate gene transcription negatively (Meister and Tuschl, 2004). Both siRNA and miRNA molecules are initially generated from dsRNAs processed by the ribonuclease III enzyme Dicer into 20-30 nucleotide duplexes (Margis et al., 2006).

In plants, whereas for the miRNAs, the related endogenous genes are transcribed into long primary microRNAs (pri-miRNAs) under the action of RNA Polymerase II (Pol II) (Wassenegger and Krczal, 2006). These pri-miRNAs, which are single-stranded and polyadenylated RNA molecules, form hairpin-like structures. Dicer-Like1 (DCL1) cleaves the pri-miRNAs, generating precursor microRNAs (pre-miRNAs) which undergo further processing by DCL1 (Zotti and Smagghe, 2015). This leads to the production of mature miRNA duplexes consisting of the active miRNA strand and its complementary strand miRNA*. The pre-miRNAs are subsequently transported to the cytoplasm through HASTY (HST), the ortholog of human exportin-5, where they undergo further processing by the cytoplasmic RNase III Dicer to produce approximately 22-nucleotide miRNAs duplexes (**Figure 1**, Left). The export of pre-miRNAs is facilitated by HST (Cambiagno et al., 2021).

For siRNAs, these RNAi-triggering dsRNAs are formed in the nucleus through several mechanisms (**Figure 1**, Right). The 21/22-nt sRNAs are primarily associated with mRNA cleavage and are involved in posttranscriptional gene silencing (PTGS) (Hamilton and Baulcombe, 1999). The 24-nt sRNAs are primarily associated with RNA-directed DNA methylation (RdDM) and transcriptional gene silencing (Henderson et al., 2006; Hamilton et al., 2015). Both metazoan and plant Dicer-Like proteins display domains such as DEAD-box, helicase-C, domain of unknown function 283 (DUF283), PIWI/Argonaute/Zwille (PAZ), RNase-III, and dsRNA-binding domain (dsRBD) domains (Parker et al., 2005). These sRNAs, which consist of 21-24 nucleotide duplexes, are subsequently incorporated into the RNA-induced silencing complex (RISC). With the RISC, they undergo unwinding (Borges and Martienssen, 2015). Following this step, an Argonaute (AGO) protein cleaves the passenger (sense) strand, while retaining the guide (antisense) strand within RISC (Kedde et al., 2007; Ketting, 2011; Gong, 2021). The guide strand of the sRNA then directs RISC to target mRNA through Watson-Crick base pairing, resulting in the cleavage of the target mRNA by the AGO protein and subsequent degradation. This degradation of the target mRNA leads to specific post-transcriptional gene silencing (Huvenne and Smagghe, 2010; Borges and Martienssen, 2015; Tyagi et al., 2019; Kaur et al., 2020). The AGO protein Nrde-3, which is necessary for nuclear RNAi in *Caenorhabditis elegans*, was identified through a genetic screen and found to reside in the cytoplasm until siRNA binding induced its translocation to the nucleus (Guang et al., 2008). The dynamic localization of RNAi factors suggests a highly regulated and adaptable system for gene regulation in cells, allowing for efficient targeting and silencing of specific genes or transcripts.

Beyond these classical miRNAs and siRNAs, other classes of RNA are continually being discovered, participating in a wide range of pathways and regulatory mechanisms. Recent research has uncovered further intricacies within the RNAi machinery. For example, the involvement of long non-coding RNAs (lncRNAs) in plant immunity, the mechanisms of lncRNA action in various stages of immunity, and different interactions between plants, microbes and insects (Wu et al., 2020; Statello et al., 2020). Additionally, a study found that aphids translocated a lncRNA into plants, which functioned as lncRNA virulence factors by enhancing aphid fecundity (Chen et al., 2020).

## 3 RNAi application in crop protection

**Figure 2:**
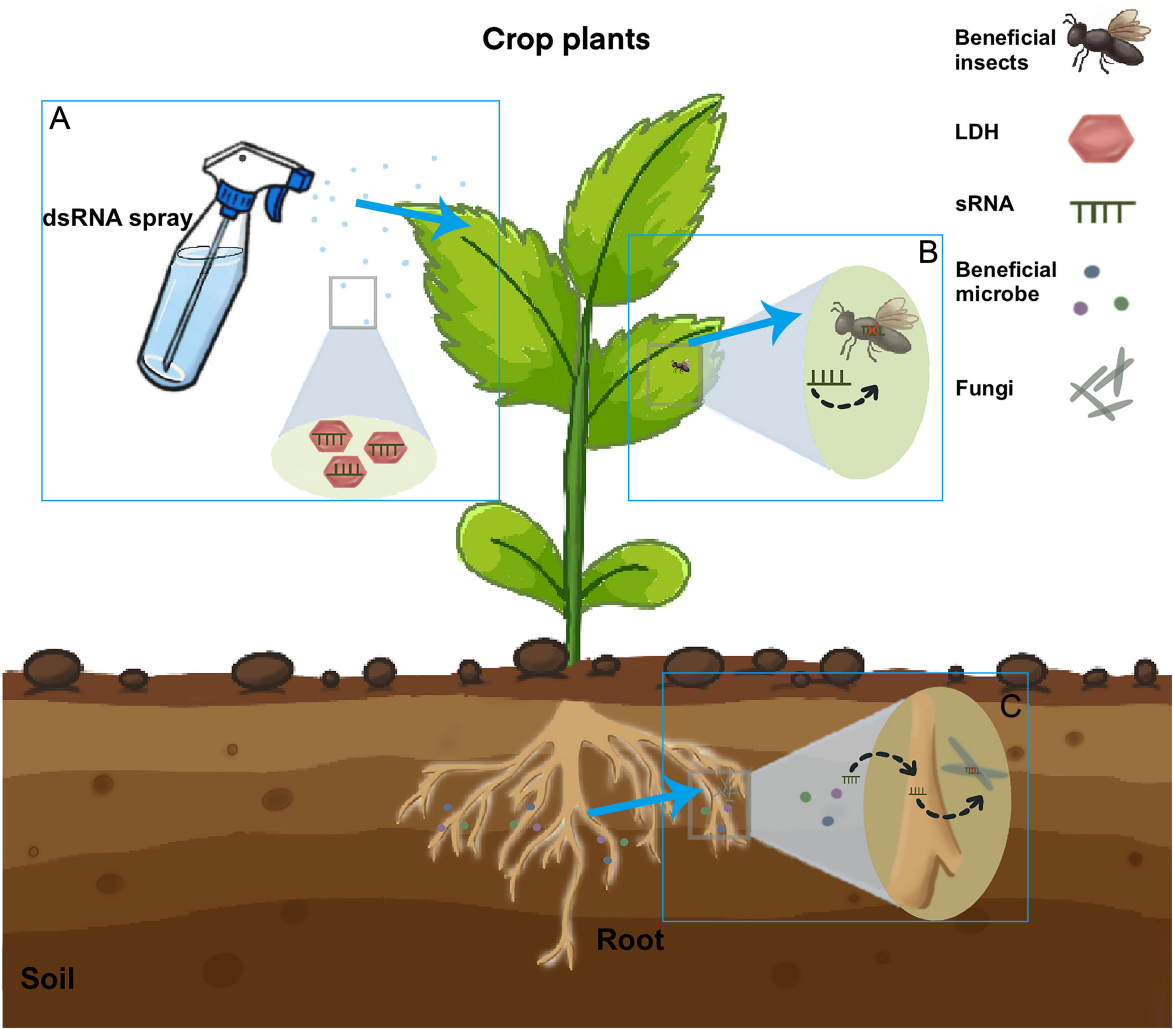
The pathway of silencing insect and fungal genes induced by sprays of sRNAs. Insects and fungi directly take up sprayed RNAs. Externally applied sRNAs are taken up by plant cells **(A)** and then transferred into insect **(B)** or Fungal cells **(C)**. In beneficial microbe process, the sRNA from the beneficial microbe would be first transferred to the host plant and then to the pathogen (Wen et al., 2023). sRNAs can be loaded onto layered double hydroxide (LDH) clay nanosheets, which are designer, non-toxic, degradable, and layered. Once loaded, the dsRNA remains on the LDH and exhibits sustained release. LDH has been developed to increase sprayed RNA stability and target delivery. Current RNA spray applications are based on mature small RNAs for spray-induced gene silencing against fungal pathogens. RNAi is also efficient for the control of plant pathogens (Joga et al., 2016). RNAi-based products can be used to suppress virus infection in pests either by administering a sugar water solution containing dsRNA or through large-scale field treatments. Future applications may be extended to mRNAs to produce inhibiting peptides inside fungi and pests.

The RNAi mechanism operates at the mRNA level through a sequence-dependent mode of action, rendering it unique in potency and selectivity compared to regular agrochemicals. One advantage of RNAi, whether through transformative or non-transformative approaches, is its potential to enable farmers to target pathogens, nematodes, and pests more specifically. The technology can be designed by using RNA sequences that match specific gene sequences in the target pathogens, nematodes, and pests, thereby minimizing harm to other species. Careful selection of unique regions of insect genes results in highly targeted effects, while avoiding unintended consequences. RNAi in crop protection can be achieved through plant transformation via PIPs, such as transgenic plants, or through non-transformative strategies employing a spray-induced gene silencing (SIGS) process. Exogenously applied dsRNA can be taken up by two means: pathogenic cells directly uptake dsRNAs to evoke RNAi in pathogens. On the other hand, exogenously applied dsRNAs can be taken up by plant cells and then transferred to interacting pathogens to induce RNAi responses. Irrespective of the delivery strategy, the use of RNA-based products to provide plant protection against pests and pathogens represents a potential alternative to conventional pesticides. Moreover, these dsRNAs can function as resistance repressors for resistant insect and weed strains. In this category of non-PIPs, dsRNA-containing end-use products (dsRNA-EPs) are anticipated to enter the market in four categories: (i) direct control agents; (ii) resistance factor repressors; (iii) development disruptors; and (iv) growth enhancers (Zotti and Smagghe, 2015; San-Miguel and Scott, 2016). This review will focus exclusively on developments related to crop protection (**Figure 2**).

The RNAi strategy, known as host-induced gene silencing (HIGS), has been successfully employed in transgenic plants to protect crops from specific insects (Head et al., 2017; Rodriguez Coy et al., 2022; Darlington et al., 2022), plant pathogens (Ray et al., 2022; McLaughlin et al., 2023), viruses (Tenllado and Díaz-Ruíz, 2001; Khalid et al., 2017), and nematodes (Sikandar et al., 2021; Morozov et al., 2019), as reviewed recently. The use of transgene-expressed dsRNA for inducing virus resistance and gene silencing in plants has proven to be effective against various viral infections and pests (Waterhouse et al., 1998). Scientists have successfully enhanced the natural defenses of plants by targeting specific genes involved in pathogen defense or insect resistance pathways, thus eliminating the need for chemical pesticides. However, approval from various regulatory agencies is required for crops that express dsRNA due to laws and regulations concerning genetically modified organisms. These factors complicate broader applications of HIGS worldwide, despite undeniable practicality and efficiency of HIGS strategies. Furthermore, the spray application of exogenous dsRNA or sRNA has initiated an era of RNAi-based fungicide strategies for controlling crop disease (Wang and Jin, 2017; Islam and Sherif, 2020). **Table 1** summarizes some examples of successful application of HIGS and SIGS through RNAi-based approaches in insects or fungi.

**Table 1:** Examples of RNA-mediated gene silencing in phytopathogenic fungi/pests via different applications to plants.

Croup	Target Organism	Target Gene(s)	Species	Application Method	Silencing Outcome	Reference
Insects	*Radopholus similis*	*Chs-2*, *Unc-87, Pat-10*, *Eng1a*	Banana	Host induced gene silencing via transgenic plants)	Reduction in nematode multiplication and root damage	Mwaka et al., 2023
	*Helicoverpa armigera*	Chitinase	Tomato and tobacco		Detrimental effects on larval growth and survival	Mamta et al., 2016
	*Bemisia tabaci*	*Trehalose-6-phosphate synthase 1 and 2*	*Nicotiana tabacum*		90% mortality and decreased the fecundity in whitefly adults	Gong et al., 2022
	*Myzus persicae*	*ATPD, ATPG*	Oilseed rape	Spraying	nanocarrier-delivered RNA pesticides affected insect survival	Ma et al., 2023
	*Sogatella furcifera*	Vacuolar-type (H+)-ATPase	Rice		More than 97% insect mortality	Ma et al., 2024
	*Sitobian avenae*	Salivary sheath protein	*Hordeum vulgare*		60% reduction in disease resistance	Biedenkopf et al., 2020
Fungi	*Aspergillus flavus*	*Alk*	Maize	Host induced gene silencing via transgenic plants)	84% reduction in aflatoxin accumulation and reduced fungal biomass	Omolehin et al., 2021
	*Fusarium oxysporum*	*Chs*	Soybean		Reduction in lesion size & fungal biomass	Kong et al., 2022
	*Fusarium graminearum*	*SGE1, PP1, STE12*	Wheat		Reduction in fungal infection structures, inhibition of DON biosynthesis	Wang et al., 2020
	*Magnaporthe oryzae*	*DES1*	Rice	Spraying	25-60% reduction in disease symptoms	Sarkar and Roy-Barman, 2021
	*Sclerotinia sclerotiorum*	*VPS51, DCTN1, SAC1*, *DCL1*, *DCL2*	Lettuce Collard green		Reduction in disease symptoms and lesion size	Qiao et al., 2021
	*Botryotiania fuckeliana*	*Chitin synthase class III, DCL 1, DCL2*	*Fragaria ananassa*		75% reduction in biomass accumulation	Islam et al., 2021

Research efforts are underway to explore non-transformative approaches to control insects, diseases, nematodes, and weeds. It is anticipated that RNAi-based products will soon be available on the market as sprays for foliar application, trunk injection, root dipping, or seed treatment to directly employ as controlling agents. Since the discovery of RNAi and its regulatory potential, scientists have been investigating diverse applications of this powerful tool in insect control. The immense potential of RNAi lies in its capacity to selectively target and suppress genes responsible for insect survival and reproduction. An important advantage of utilizing RNAi in insect control is its distinct mode of action in comparison to traditional chemical pesticides (Kupferschmidt, 2013; Cagliari et al., 2019). RNAi-based products can be designed to selectively target particular pests while sparing non-target species. This targeted approach not only minimizes environmental impact but also mitigates the risk of pest resistance development.

dsRNA-based products, such as RNAi therapeutics or RNAi-based pesticides, utilize this mechanism to selectively suppress disease-causing genes or pest-specific genes. By designing dsRNA molecules that are complementary to the target gene’s mRNA, researchers can effectively shut down the expression of the target gene, potentially leading to the treatment of genetic disorders or enhanced crop protection. One of the earliest studies in the use of sprayable RNA molecules to control insect pests involved the application of siRNA, which led to effective RNAi silencing in the diamondback moth (*Plutella xylostella*). When larvae were fed with *Brassica* spp. leaves sprayed with chemically synthesized siRNAs targeting the acetylcholine esterase gene *AchE2*, mortality rates of about 60% were observed (Gong et al., 2013).

Moreover, the use of RNA to target pathogen resistance to conventional fungicides is currently in development (Song et al., 2018). *Botrytis cinerea* transfers small RNAs into plant cells, which then bind to the plant’s AGO1 to suppress host genes essential for plant immunity (Weiberg et al., 2013). This application is sprayed onto the surface of fruits (such as tomato, strawberry, and grape), and vegetables (such as lettuce and onion), resulting in a significant inhibition of grey mold disease development (Wang et al., 2016).

Recent advances in nanoparticle technology have significantly improved the potential applications for plant protection. To address issues related to dsRNA stability, double-layered hydroxide (LDH) nanoparticles were developed and combined with dsRNA molecules to produce “BioClay” (Mitter et al., 2017; Dubrovina and Kiselev, 2019; Yong et al., 2021; Jha et al., 2023). Nanoparticles are particles with diameters ranging from 1 to 100 nanometres (nm), with high stability and transport in plants (Cheng et al., 2024). LDH also degrades safely under mildly acidic conditions, thereby minimizing risk of the excessive persistence of dsRNA in the environment (Abd El-Monaem et al., 2023). Foliar spraying of LDH-dsRNA could disrupt *Bemisia tabaci* at multiple whitefly developmental stages by enhancing the delivery of dsRNA to cotton leaves (Jain et al., 2022). Additionally, optimizing nanostructures for agricultural use enhances the stability of RNA-based treatments, boosting control effectiveness and minimizing environmental impact (Wang et al., 2023).

Up to now, RNA-based molecules (dsRNA or siRNA) commonly utilized in insect and disease management studies have been expensive synthetic molecules or have been produced through time-consuming, laborious procedures. To address the limitations of these methods, the potential of delivering dsRNA expressed in bacteria has been investigated, providing an alternative method for large-scale target gene screening. For instance, the *Escherichia coli* HT115 (DE3) strain has been used to produce large quantities of dsRNA due to its lack of the enzyme RNase III which degrades dsRNA (Ahn et al., 2019; Figueiredo-Prates et al., 2023). Nanomaterials derived from plant viruses offer a promising alternative to synthetic nanoparticles. Unlike synthetic nanoparticles, plant viruses offer a higher environmental biocompatibility and degradability, yet they are exceptionally robust in the harsh environment. And most importantly, they are non-infections to mammals. Indeed, some of the most widely used plant viruses have been cowpea mosaic virus (CPMV) (Beatty and Lewis, 2019; Ortega-Rivera et al., 2021) and tobacco mild green mosaic virus (TMGMV) (Caparco et al., 2023). Practical applications of RNAi have rapidly advanced with transgene-expressed dsRNA employed not only for inducing virus resistance and gene silencing in plants but also for enhancing crop traits such as yield improvement and nutritional enhancement. Although the precise mechanism of external RNA recognition, uptake, and transport is yet to be determined, recent studies demonstrated that exogenous RNA application is a promising strategy for the regulation of plant properties, indicating the need for further research.

## 4 Overcome barriers to foliar uptake of sRNAs

Indeed, efficient methods to facilitate the uptake of exogenous dsRNA for RNAi-mediated crop protection are essential for practical applications. In recent years, there has been a growing interest in the market for dsRNA, promoting both established companies and startups to focus on enhancing production efficiency and developing stable delivery systems. With the potential of dsRNA for crop protection, many companies and academic researchers are exploring cost-efficient methods for large-scale dsRNA production. It is speculated that externally applied synthetic dsRNA and siRNA may enter plant tissues and cells through natural mechanisms similar to those used by extracellular nucleic acids from plant microbial pathogens, insects, or viruses (Tatematsu et al., 2018; Dubrovina and Kiselev, 2019; Dad et al., 2021; Liu G. et al., 2021). However, the existing literature offers limited insight into the natural mechanisms responsible for the recognition, uptake, and translocation of exogenous nucleic acids in plant tissues.

Environmental risks associated with chemical and microbial pesticides are typically evaluated using a tiered approach (Pathak et al., 2022). This approach often involves testing the maximum hazard dose of known environmental exposure concentration using non-target indicator species from different ecological guilds such as pollinators, predators, and parasitoids (Lundgren and Duan, 2013). Predicting toxic effects and designing maximum hazard assays for the numerous potentially exposed organisms is a challenging task. It has been reported that foliar applied transgene-encoding dsRNA was detectable in RNA probes obtained from treated *Arabidopsis* leaves at 1 day and 7 days post-treatment, but its presence sharply decreased after 14 days (Lundgren and Duan, 2013). The “RNAgri” agricultural industry has developed microbial fermentation technology for large-scale production of dsRNA, utilizing a protein to bind the desired RNAs and protect them against degradation. The resulting dsRNA products are considered safer to use and more stable than naked dsRNA (Numata et al., 2014). Additionally, bacterial minicells have shown promise as a technology for both the production and encapsulation of dsRNA (Islam et al., 2021). If successful, this technology could provide improved shielding and slow, sustained release of dsRNA for agricultural purposes under open-field conditions. It is important to note that siRNAs might exhibit off-target binding in the genome of non-target species. However, considerations for microbial production of dsRNA include potential by-products from fermentation and the additional concern of GMOs (Figueiredo-Prates et al., 2023). In addition to the standard quality control measures aimed at ensuring dsRNA purity, bacterial production systems require meticulous attention to exclude potential contaminants and living GMOs. Longer dsRNA (>200 nt) yields many siRNAs after cleavage, enhancing RNAi response and reducing resistance (Darlington et al., 2022). Transgenic plants, with continuous dsRNA, increase selection pressure and resistance. RNAi resistance may come from reduced cellular uptake (Khajuria et al., 2018), mRNA mutations, RNAi suppressor production (Tayler et al., 2019), target gene overexpression, silencing machinery gene downregulation (Timani et al., 2023), increased nuclease activity, or behavioral changes (Spit et al., 2017; Kadoić-Balaško et al., 2020). Recently, cell-free platforms for dsRNA synthesis have been established, allowing for cost-effective, GMO-free production of significant quantities of dsRNA suitable for use in agricultural applications. The efficiency of RNAi naturally varies among the target species, life stage, and delivery strategy. Choosing appropriate combination of these factors can significantly expedite research and conservation of resources. Regardless of the delivery strategy or target species, the successful implementation of a non-transformative RNAi strategy hinges on the identification of unique regions within essential target genes. This ensure that even minor changes in expression levels will elicit substantial consequences.

### 4.1 Uptake efficiency

Broad translational success of RNAi technology depends on effective delivery approaches. In order to access the plant RNAi machinery for small RNA production, dsRNA needs to penetrate the cytoplasm. The plasma membrane, composed of different components than the cell wall, serves as a highly selective barrier that restricts the entry of extracellular particles.

The cell membrane is a negatively charged lipid bilayer with transmembrane channels and transporters that regulate the movement of small molecules across membranes through active transport, osmosis, and diffusion. Recent reports emphasize the importance of extracellular vesicles (EVs) in facilitating the transport of various plant defense and virulence factors between the plant and the pathogen (Bahar et al., 2016; Mordukhovich and Bahar, 2017; Yang et al., 2020; Tran et al., 2022). EVs are heterogenous phospholipid bilayer membrane-bound spherical structures that carry biologically active cargo such as liposomes, proteins, and nucleic acids (Liu G. et al., 2021). They have been implicated in cell-to-cell communication and biomolecules transfer (Colombo et al., 2014; Mathieu et al., 2019; Gurunathan et al., 2021). The topical application of exogenous nanoparticle-dsRNA complexes on plants can also improve the absorption efficiency of dsRNA into pathogens. Recent advances in RNA-based products have utilized liposomes and synthetic spherical lipid-based nanoparticles for sRNA delivery (Yan et al., 2021; Komarova et al., 2023; Qiao et al., 2023).

### 4.2 Stability on plant surfaces

RNAi efficacy is impacted by the persistence and stability of topically applied dsRNA before entering the plant. As foliar uptake of dsRNA is not instant, longer leaf-surface retention ensures a stable supply of dsRNA. Environmental elements such as rain, ultraviolet (UV) radiation, and microbial degradation can degrade dsRNA and diminish its effectiveness. Therefore, the development of a delivery system that shields dsRNA from degradation is crucial for practical field applications. Recent studies have revealed that formulating dsRNA with nanocarriers can protect it from UV and nuclease degradation (Schwartz et al., 2020; Zhang et al., 2022; Ijaz et al., 2023). Additionally, the use of nanoparticles has also demonstrated increased persistence of sprayed dsRNA on leaf surfaces even after rinsing (Vatanparast and Kim, 2017; Nitnavare et al., 2021). These approaches showed that when dsRNA is complexed with layered BioClay, it tends to remain on leaves, while unprotected dsRNA is readily washed off. Furthermore, nanoparticles have the potential to improve foliar and cellular uptake of sprayed dsRNA (Golestanipour et al., 2018; Wu et al., 2023; Mat-Jalaluddin et al., 2023). For instance, it has been demonstrated that BioClay delivered dsRNA can prolong protection against *B. cinerea* on tomato leaves and fruit, as well as on mature chickpea plants (Niño-Sánchez et al., 2022). Specifically, BioClay increased the protection duration from 1 to 3 weeks on tomato leaves and from 5 to 10 days on tomato fruits when compared with naked dsRNA.

In summary, while RNAi as a foliar spray exhibits potential for insect control, there are challenges to be addressed for successful field applications. Continued research and development efforts are essential to overcome these challenges and fully harness the potential of this technology in agriculture.

### 4.3 Delivery scalability

Nanotechnology-mediated RNAi provides new approaches for the control strategies of plant diseases and insect pests. Delivery scalability must be considered when dsRNA is exogenously applied to crops. Utilizing RNAi as a foliar spray in large-scale agricultural fields necessitates efficient and cost-effective delivery systems. The successful cellular uptake and subsequent initiation of target gene silencing by exogenous dsRNA are influenced by various factors that can influence their effectiveness (Das and Sherif, 2020). One such factor is the length of the dsRNA utilized. Studies have indicated that shorter dsRNA tend to be more effective in gene silencing compared to longer ones, as they can more readily penetrate cells and interact with their target genes (Numata et al., 2014; Dalakouras et al., 2016; Dubrovina and Kiselev, 2019). sRNAs can move short and long distances within plant cells. Primary siRNAs can spread over short distances (10-15 cells) through the symplastic route without producing secondary siRNAs (Han et al., 2019). Systemic spreading of RNA silencing via the phloem has been reported in studies using plant transformation (David-Schwartz et al., 2008; Liu and Chen, 2018). Long-distance movement or systemic silencing is phloem-mediated and requires the amplification of silencing signals by RNA-dependent RNA plolymerases (RDRPs) (Cuperus et al., 2010; Bally et al., 2016; Dalakouras et al., 2018; Uslu et al., 2020). It has been documented in several studies that the phloem-mediated transport of systemic sRNA signals from source to sink (Qiao et al., 2018; Yan and Ham, 2022). Furthermore, the concentration of applied dsRNA also plays a crucial role in its efficacy. Higher concentrations may enhance cellular uptake, but excessive amounts can potentially cause off-target effects or even toxicity (Willow et al., 2021). Therefore, finding an optimal concentration is essential for achieving successful gene silencing without adverse effects.

It is worth noting that the effectiveness of exogenous dsRNA-induced RNAi in plant-microbe interactions can vary depending on factors such as the delivery method, targeting strategy, and the specific organisms involved. Ongoing research aims to further optimize and understand the potential of this approach for sustainable agriculture and crop protection (Yan et al., 2020).

## 5 Mitigation and avoidance of risks associated with RNAi-based products

RNAi is an emerging technology that offers new opportunities for innovative insect control strategies and the development of desired traits in genetically modified plants. The next generation of RNAi-based products will harness dsRNA to induce gene suppression in insect species, either through *in planta* production or application via plant spraying. Regulatory agencies for biotechnological products, such as the U.S. Environmental Protection Agency (US-EPA) and the European Food Safety Authority (EU-EFSA), have extensive experience in assessing ecological risk associated with newly introduced PIPs, the unique mode of action of dsRNA may necessitate a distinct assessment framework, as indicated by several years of crop assessments (Devos et al., 2014; Papadopoulou et al., 2020). To fully harness the potential of RNAi-based products, effective communication among agricultural consumers, RNAi-based product users (farmers), RNA pesticides producers, and public institutions will be essential to ensure accurate information exchange and mutual understanding. This should be firmly rooted in scientific evidence pertaining to RNAi-based products. Additionally, the implementation of appropriate management, oversight, and legislation, along with safety evaluation and approval processes, will be imperative for the successful adoption and regulation of RNAi-based products.

### 5.1 Regulatory concerns

The utilization of RNAi-based products in agriculture may provoke regulatory concerns regarding their safety, potential off-target effects, and long-term impacts on ecosystems (Paces et al., 2017; Christiaens et al., 2018; Dávalos et al., 2019). Adhering to regulatory guidelines and requirements is vital for the development and approval of RNAi-based products. Close collaboration with regulatory authorities throughout the development process helps early identify potential risks and effective resolution (Arpaia et al., 2020). It is important to consider, for the risk assessment purposes, that RNAi-based products might take longer to demonstrate efficacy compared to conventional pesticides (Romeis and Widmer, 2020; Fletcher et al., 2020). Globally, the legislative status of dsRNA pesticides varies, which reflects diverse regulatory approaches.

In the USA, biochemical pesticides still need to be registered by the US EPA before manufacture, transport, and sale (Leahy et al., 2014). The EPA approves them under the Federal Insecticide, Fungicide, and Rodenticide Act (FIFRA) and the Federal Food, Drug, and Cosmetic Act (FFDCA), basing the approval on a risk/benefit standard. While no specific data requirements for sprayable or externally applied dsRNA-based pesticides are available, the EPA requires that the active ingredient as well as the final product must be evaluated (Wozniak et al., 2024).

Australia has an advanced and efficient agricultural industry, focusing on developing innovative systems through research and design to enhance food production and sustainability. In terms of RNAi-based products, Australia has been a pioneer in establishing a legal structure for approving these crop protection products. By October 8, 2019, topically applied dsRNA-based products for protecting plants against pests (insects, fungi, and viruses) are defined as agricultural chemical products. Previously, the office of the gene technology regulator (OGTR) and the Australian Pesticides and Veterinary Medicines Authority (APVMA) regulated these products. However, the OGTR’s review indicated that applying RNA to an organism for the induction of temporary RNAi is not GMOs. So, SIGS-applications are not under OGTR regulation and should be regulated based on risk. Currently, there are no specific guidelines regarding data requirements for the registration of RNAi-based agricultural products. However, at a minimum, data related to chemistry, manufacturing, human health, worker health and safety, environmental fate and toxicity, efficacy, and crop safety are required. In Australia, the APVMA will keep providing regulatory supervision for topically applied RNAi-based products. And the APVMA must be ensure that the safety, trade, and efficacy requirements related to the specific active ingredient or product are fulfilled. On February 9, 2021, the Food Standards Australia New Zealand (FSANZ) approved the RNAi-based herbicide-tolerant and insect-resistant corn product DP23211 for food. This transgenic corn simultaneously expresses the ds*DvSSJ1* and IPD072Aa proteins for the control of corn rootworms (*Diabrotica* spp.). In addition, multiple transgenic plants based on RNAi technology have been approved for commercial cultivation. In 2014, JR Simplot’s InnateÔ (SPS-ØØE12-8 (E12)) potato was approved for cultivation in the United States and subsequently approved in many countries such as Malaysia, Canada, Mexico, Japan, Australia, and New Zealand (James, 2014).

In the EU, any plant protection product (PPP), which is a pesticide safeguarding crops or other valuable plants, must receive authorization before being place on market. The legal framework for this process is defined in Regulation (EC) No. 1107/2009 (EC, 2009). The authorization process consists of approving the active substance and subsequently authorizing the PPP. The authorization of a PPP is conditional upon the approval of the active substance by the EU Commission, based on a risk assessment conducted by the EFSA. A PPP containing an approved active substance is then assessed and authorized by the Member States (MS). To streamline the authorization process, the EU is divided into three zones, the Northern, Central, and Southern (EC, 2009). The risk assessment of a PPP is conducted by one Member State for the entire zone, with the Additional Member States in the same zone must accepting the results and decision of the assessing state. However, they can make claims based on national ecological or agricultural specificities to determine their risk management options. The European Medicines Agency (EMA) mandates an extensive risk assessment process for novel therapeutic approaches such as RNA interference (Hines et al., 2020; Talap et al., 2021). This assessment includes evaluating potential off-target effects on non-target genes that could lead to adverse events. RNAi is mechanism that relies on sequence homology. Several studies have indicated that siRNA is not always specific and can lead to off-target effects (Carthew and Sontheimer, 2009). RNAi have the potential to impact unintended organisms when the target gene share homologous sequences with non-target organisms, thus resulting in unintended environmental consequences and affecting beneficial organisms. The efficiency of RNAi as a foliar spray is influenced by several factors. First, the selection of target gene is critical. Identifying genes that are essential for insect survival or reproduction increases the likelihood of successful RNAi-mediated suppression (Ghosh et al., 2017; Mamta and Rajam, 2017). Second, it is important to design dsRNA molecules that are specific to the target gene and can be efficiently taken up by the pests.

To ensure the safety, efficacy, and quality of RNAi-based products, it is essential for companies to strictly adhere to regulatory guidelines. Distinguishing regulatory studies as either risk-driven or advocacy-driven would help dispel the public’s misconception that extensive and intricate regulations for products developed through modern breeding techniques solely aimed at addressing higher safety risks (Auer and Frederick, 2009; Qaim, 2020). This suggestion assumes that assessing potential safety concerns is more important than promoting consumer acceptance of these technologies and products. Utilizing bioinformatics could assist in identifying off-target sequences and potential effects in non-target species. These guidelines are established by regulatory authorities such as the Food and Drug Administration (FDA) in the United States or EMA in Europe (Hokaiwado et al., 2008; Mohr and Perrimon, 2012; Setten et al., 2019). Compliance with these guidelines allows companies to demonstrate that their products meet all necessary standards before they can be approved for use. For instance, it was reported that siRNA resulting from Dicer-2 processing can have a variable length (20-22 nt) in different insect species (Christiaens et al., 2018).

### 5.2 Post market surveillance

After a product receives market approval, continuous monitoring and surveillance are necessary to identify any potential safety concerns that may arise post-commercialization. Encouraging professionals to report any adverse events helps ensure timely identification and mitigation of risks. When assessing the environmental risks associated with the use of dsRNA-based pesticides, it is crucial to consider the distribution, stability, and persistence of dsRNA in the environment following product application (OECD, 2020). It has shown a rapid decline in the concentration of foliar-applied dsRNA under field conditions with a 95% reduction after 3 days (Bachman et al., 2020). Recent studies have indicated that the decrease of dsRNA in soil is attributed to both adsorption to soil and chemical and microbial degradation (Dubelman et al., 2014; Parker et al., 2019). The flag bearer here is the global giant Monsanto whose brand “Biodirect” is already developing RNA-based biopesticides to control pests, followed by other multinationals such as Bayer, Syngenta, and others (Abdellatef et al., 2021).

While RNAi-based technologies offer evident safety advantages compared to numerous current crop protection products, unintended impacts on human health (OECD, 2023) and the environment cannot be completely ruled out (OECD, 2020). It is important for researchers and regulatory agencies to continue studying and monitoring the potential effects of RNAi-based technologies order to ensure their safe use in agriculture (Auer and Frederick, 2009). Additionally, public awareness and education about these technologies can help address any concerns or misconceptions surrounding their impact on human health. It is considered unlikely for dsRNA to pose a significant risk to human health in a sequence-specific manner, because of the difficulty in achieving successful systemic delivery (Chen et al., 2018). Systemic exposure following consumption of plants containing dsRNA that mediate RNAi is limited in higher organisms by extensive physical and biochemical barrier (Jay et al., 2013). The approach involves comparing regions of the dsRNA with gene orthologs in databases for non-target species in different agroecosystems, to avoid off-target effects as siRNA. Non-coding RNAs in biological fluids are unstable due to enzymes and kidney removal (Moreno et al., 2021). Environmental dsRNA without protection would have similar issues. Introduced dsRNA must be similar enough to endogenous transcripts for degradation, but their impact may still be minimal due to delivery constraints. RNAi-based insect pest control is not extremely effective in various pests because the dsRNA degrades quickly and the insects don’t take it up or process it efficiently. In addition, dsRNA can be degraded by nucleases in nucleic acid digestion in the insect midgut guts as has for example been reported for Liu et al. (2012). To counter unintended impacts on closely related beneficial species, an understanding of the setting in which the RNAi technology will be applied is key. Overall, a balanced approach that considers both the benefits and potential risks of RNAi-based technologies is necessary for their responsible implementation in crop protection.

## 6 Conclusions

In conclusion, the utilization of exogenous dsRNA-induced RNAi technology holds great potential for revolutionizing various aspects of agriculture and crop management. By harnessing this innovative approach, we can pave the way for more eco-friendly and sustainable practices in gene regulation. By targeting genes related to plant diseases, exogenous dsRNA-induced RNAi offers a promising solution for combating plant pathogens, potentially reducing reliance on chemical pesticides and minimizing their harmful effects on human health and the environment (OECD, 2020; 2023). This technology also benefits crop improvement by selectively targeting specific genes involved in desirable traits such as drought tolerance or insect resistance, thus accelerating breeding programs, and developing new varieties with enhanced resilience and productivity (Jothi et al., 2023). However, before exogenous dsRNA-induced RNAi can be widely adopted in agricultural practices at large-scale levels such as greenhouses and fields, several essential considerations need to be addressed. One critical aspect is production technologies-optimizing methods for synthesizing quantities of high-quality dsRNA molecules efficiently will be essential to meet the demands of commercial applications. Additionally, cost-effectiveness plays a vital role in determining whether this technology becomes economically viable on a larger scale. Research efforts should focus on finding ways to reduce production costs while maintaining efficacy. Enhancing the stability of dsRNA is critically significant because it directly impacts the effectiveness of applications. In challenging environmental conditions, dsRNA is susceptible to degradation, resulting in diminished efficacy. Advancements in dsRNA delivery mechanisms are essential. Currently, the available methods exhibit low efficiency and poor targeting capabilities. In agricultural applications, encapsulating dsRNA within liposomes can facilitate better adherence to plant foliage and enable timely release, thus allowing for precise action against pests and enhancing pest management efficiency. The development of novel carriers or the optimization of existing delivery system parameters can further enhance the effectiveness of dsRNA.

## 7 Future directions

The United Nations Environment Programme (UNEP) and the Food and Agriculture Organization (FAO) have recognized the crucial necessity of rehabilitating degraded ecosystems. They claim that the required restoration efforts are indispensable for attaining sustainability goals, including food and water supply security, biodiversity preservation, poverty alleviation, and climate change mitigation. Comprehensive risk assessments are necessary to ensure environmental safety when deploying exogenous dsRNA-induced RNAi technology extensively. When adequate conservation biocontrol strategies are implemented in combination with RNAi technology, the effectiveness of sustainable crop protection can be greatly enhanced. This is especially significant considering that RNAi products have relatively lower environmental risks compared to traditional pesticides.The U.S. EPA has officially approved the innovative RNA-based biopesticide Ledprona by Greenlight Biosciences to combat the increasingly severe destructive pest, the Colorado potato beetle (CPB, *Leptinotarsa decemlineata*) (Kadoić-Balaško et al., 2020; Rodrigues et al., 2021). The EPA emphasizes that RNA-based biopesticides provide farmers with additional tools to address the challenges of climate change and contribute to resistance management. In summary, while there are still challenges ahead regarding production technologies and cost-effectiveness issues surrounding exogenous dsRNA-induced RNAi technology’s implementation in large-scale agricultural settings like greenhouses and open fields, continued research efforts will play a pivotal role in realizing its potential to achieve more sustainable farming practices worldwide.

## References

[B1] Abdellatef E. Kamal N. M. Tsujimoto H. (2021). Tuning beforehand: a foresight on RNA interference (RNAi) and in *vitro*-derived dsRNAs to enhance crop resilience to biotic and abiotic stresses. Int. J. Mol. Sci. 22, 7687. doi: 10.3390/ijms22147687 34299307 PMC8306419

[B2] Abd El-Monaem E. M. Elshishini H. M. Bakr S. S. El-Aqapa H. G. Hosny M. Andaluri G.. (2023). A comprehensive review on LDH-based catalysts to activate persulfates for the degradation of organic pollutants. NPJ Clean Water 6, 34. doi: 10.1038/s41545-023-00245-x

[B3] Ahn S. J. Donahue K. Koh Y. Martin R. R. Choi M. Y. (2019). Microbial-based double-stranded RNA production to develop cost-effective RNA interference application for insect pest management. Int. J. Insect Sci. 11, 1179543319840323. doi: 10.1177/1179543319840323 31040730 PMC6482651

[B4] Ambros V. (2004). The functions of animal microRNAs. Nature 431, 350–355. doi: 10.1038/nature02871 15372042

[B5] Arpaia S. Christiaens O. Giddings K. Jones H. Mezzetti B. Moronta-Barrios F.. (2020). Biosafety of GM crop plants expressing dsRNA: data requirements and EU regulatory considerations. Front. Plant Sci. 11. doi: 10.3389/fpls.2020.00940 PMC7327110 32670333

[B6] Auer C. Frederick R. (2009). Crop improvement using small RNAs: applications and predictive ecological risk assessment. Trends Biotechnol. 27, 644–651. doi: 10.1016/j.tibtech.2009.08.005 19796832

[B7] Bachman P. Fischer J. Song Z. Urbanczyk-Wochniak E. Watson G. (2020). Environmental fate and dissipation of applied dsRNA in soil, aquatic systems, and plants. Front. Plant Sci. 11. doi: 10.3389/fpls.2020.00021 PMC7016216 32117368

[B8] Bahar O. Mordukhovich G. Luu D. D. Schwessinger B. Daudi A. Jehle A. K.. (2016). Bacterial outer membrane vesicles induce plant immune responses. Mol. Plant Microbe Interact. 29, 374–384. doi: 10.1094/MPMI-12-15-0270-R 26926999

[B9] Bally J. McIntyre G. J. Doran R. L. Lee K. Perez A. Jung H.. (2016). In-plant protection against *helicoverpa armigera* by production of long hpRNA in chloroplasts. Front. Plant Sci. 7. doi: 10.3389/fpls.2016.01453 PMC5040858 27746796

[B10] Bartel D. P. (2009). MicroRNAs: target recognition and regulatory functions. Cell 136, 215–233. doi: 10.1016/j.cell.2009.01.002 19167326 PMC3794896

[B11] Beatty P. H. Lewis J. D. (2019). Cowpea mosaic virus nanoparticles for cancer imaging and therapy. Adv. Drug Delivery Rev. 145, 130–144. doi: 10.1016/j.addr.2019.04.005 31004625

[B12] Biedenkopf D. Will T. Knauer T. Jelonek L. Alexandra-Charlotte-Ursula F. Busche T.. (2020). Systemic spreading of exogenous applied RNA biopesticides in the crop plant Hordeum vulgare. ExRNA 2, 12. doi: 10.1186/s41544-020-00052-3

[B13] Borges F. Martienssen R. A. (2015). The expanding world of small RNAs in plants. Nat. Rev. Mol. Cell Biol. 16, 727–741. doi: 10.1038/nrm4085 26530390 PMC4948178

[B14] Cagliari D. Dias N. P. Galdeano D. M. Dos-Santos E. Á. Smagghe G. Zotti M. J. (2019). Management of pest insects and plant diseases by non-transformative RNAi. Front. Plant Sci. 10. doi: 10.3389/fpls.2019.01319 PMC6823229 31708946

[B15] Cambiagno D. A. Giudicatti A. J. Arce A. L. Gagliardi D. Li L. Yuan W.. (2021). HASTY modulates miRNA biogenesis by linking pri-miRNA transcription and processing. Mol. Plant 14, 426–439. doi: 10.1016/j.molp.2020.12.019 33385584

[B16] Caparco A. A. Gonzalez-Gamboa I. Hays S. S. Pokorski J. K. Steinmetz N. F. (2023). Delivery of nematicides using TMGMV-derived spherical nanoparticles. Nano Lett. 23, 5785–5793. doi: 10.1021/acs.nanolett.3c01684 37327572

[B17] Carthew R. W. Sontheimer E. J. (2009). Origins and mechanisms of miRNAs and siRNAs. Cell 136, 642–655. doi: 10.1016/j.cell.2009.01.035 19239886 PMC2675692

[B18] Chen X. Mangala L. S. Rodriguez-Aguayo C. Kong X. Lopez-Berestein G. Sood A. K. (2018). RNA interference-based therapy and its delivery systems. Cancer Metastasis Rev. 37, 107–124. doi: 10.1007/s10555-017-9717-6 29243000 PMC5898634

[B19] Chen Y. Singh A. Kaithakottil G. G. Mathers T. C. Gravino M. Mugford S. T.. (2020). An aphid RNA transcript migrates systemically within plants and is a virulence factor. Proc. Natl. Acad. Sci. U.S.A. 117, 12763–12771. doi: 10.1073/pnas.1918410117 32461369 PMC7293609

[B20] Cheng X. Zhou Q. Xiao J. Qin X. Zhang Y. Li X.. (2024). Nanoparticle LDH enhances RNAi efficiency of dsRNA in piercing-sucking pests by promoting dsRNA stability and transport in plants. J. Nanobiotechnol. 22, 544. doi: 10.1186/s12951-024-02819-4 PMC11378424 39237945

[B21] Chi X. Wang Z. Wang Y. Liu Z. Wang H. Xu B. (2023). Cross-kingdom regulation of plant-derived miRNAs in modulating insect development. Int. J. Mol. Sci. 24, 7978. doi: 10.3390/ijms24097978 37175684 PMC10178792

[B22] Christiaens O. Dzhambazova T. Kostov K. Arpaia S. Joga M. R. Urru I. (2018). Literature review of baseline information on RNAi to support the environmental risk assessment of RNAi-based GM plants. EFSA Support 15, EN–1424. doi: 10.2903/sp.efsa

[B23] Colombo M. Raposo G. Théry C. (2014). Biogenesis, secretion, and intercellular interactions of exosomes and other extracellular vesicles. Annu. Rev. Cell Dev. Biol. 30, 255–289. doi: 10.1146/annurev-cellbio-101512-122326 25288114

[B24] Cuperus J. T. Carbonell A. Fahlgren N. Garcia-Ruiz H. Burke R. T. Takeda A.. (2010). Unique functionality of 22-nt miRNAs in triggering RDR6-dependent siRNA biogenesis from target transcripts in Arabidopsis. Nat. Struct. Mol. Biol. 17, 997–1003. doi: 10.1038/nsmb.1866 20562854 PMC2916640

[B25] Dad H. A. Gu T. W. Zhu A. Q. Huang L. Q. Peng L. H. (2021). Plant exosome-like nanovesicles: emerging therapeutics and drug delivery nanoplatforms. Mol. Ther. 29, 13–31. doi: 10.1016/j.ymthe.2020.11.030 33278566 PMC7791080

[B26] Dalakouras A. Jarausch W. Buchholz G. Bassler A. Braun M. Manthey T.. (2018). Delivery of hairpin RNAs and small RNAs into woody and herbaceous plants by trunk injection and petiole absorption. Front. Plant Sci. 9. doi: 10.3389/fpls.2018.01253 PMC6120046 30210521

[B27] Dalakouras A. Wassenegger M. McMillan J. N. Cardoza V. Maegele I. Dadami E.. (2016). Induction of silencing in plants by high-pressure spraying of in *vitro*-synthesized small RNAs. Front. Plant Sci. 7. doi: 10.3389/fpls.2016.01327 PMC5003833 27625678

[B28] Darlington M. Reinders J. D. Sethi A. Lu A. L. Ramaseshadri P. Fischer J. R.. (2022). RNAi for western corn rootworm management: lessons learned, challenges, and future directions. Insects 13, 57. doi: 10.3390/insects13010057 35055900 PMC8779393

[B29] Das P. R. Sherif S. M. (2020). Application of exogenous dsRNAs-induced RNAi in agriculture: challenges and triumphs. Front. Plant Sci. 11. doi: 10.3389/fpls.2020.00946 PMC7330088 32670336

[B30] Dávalos A. Henriques R. Latasa M. J. Laparra M. Coca M. (2019). Literature review of baseline information on non-coding RNA (ncRNA) to support the risk assessment of ncRNA-based genetically modified plants for food and feed. EFSA Support 16, EN–1688. doi: 10.2903/sp.efsa

[B31] David-Schwartz R. Runo S. Townsley B. Machuka J. Sinha N. (2008). Long-distance transport of mRNA via parenchyma cells and phloem across the host-parasite junction in *Cuscuta* . New Phytol. 179, 1133–1141. doi: 10.1111/j.1469-8137.2008.02540.x 18631294

[B32] Devos Y. Aguilera J. Diveki Z. Gomes A. Liu Y. Paoletti C.. (2014). EFSA’s scientific activities and achievements on the risk assessment of genetically modified organisms (GMOs) during its first decade of existence: looking back and ahead. Transgenic Res. 23, 1–25. doi: 10.1007/s11248-013-9741-4 23963741

[B33] Dubelman S. Fischer J. Zapata F. Huizinga K. Jiang C. Uffman J.. (2014). Environmental fate of double-stranded RNA in agricultural soils. PloS One 9, e93155. doi: 10.1371/journal.pone.0093155 24676387 PMC3968063

[B34] Dubrovina A. S. Kiselev K. V. (2019). Exogenous RNAs for gene regulation and plant resistance. Int. J. Mol. Sci. 20, 2282. doi: 10.3390/ijms20092282 31072065 PMC6539981

[B35] EC (2009). Regulation (EC) no 1107/2009 of the European parliament and of the council of 21 October 2009 concerning the placing of plant protection products on the market and repealing council directives 79/117/EEC and 91/414/EEC. (Luxembourg: European Union). 309, 1–50.

[B36] Feng J. X. Riddle N. C. (2020). Epigenetics and genome stability. Mamm. Genome 31, 181–195. doi: 10.1007/s00335-020-09836-2 32296924

[B37] Figueiredo-Prates L. H. Merlau M. Rühl-Teichner J. Schetelig M. F. Häcker I. (2023). An optimized/scale up-ready protocol for extraction of bacterially produced dsRNA at good yield and low costs. Int. J. Mol. Sci. 24, 9266. doi: 10.3390/ijms24119266 37298215 PMC10253028

[B38] Fletcher S. J. Reeves P. T. Hoang B. T. Mitter N. (2020). A perspective on RNAi-based biopesticides. Front. Plant Sci. 11. doi: 10.3389/fpls.2020.00051 PMC7028687 32117388

[B39] Ghosh S. K. Hunter W. B. Park A. L. Gundersen-Rindal D. E. (2017). Double strand RNA delivery system for plant-sap-feeding insects. PloS One 12, e0171861. doi: 10.1371/journal.pone.0171861 28182760 PMC5300277

[B40] Golden D. E. Gerbasi V. R. Sontheimer E. J. (2008). An inside job for siRNAs. Mol. Cell. 31, 309–312. doi: 10.1016/j.molcel.2008.07.008 18691963 PMC2675693

[B41] Golestanipour A. Nikkhah M. Aalami A. Hosseinkhani S. (2018). Gene delivery to tobacco root cells with single-walled carbon nanotubes and cell-penetrating fusogenic peptides. Mol. Biotechnol. 60, 863–878. doi: 10.1007/s12033-018-0120-5 30203379

[B42] Gong P. (2021). Structural basis of viral RNA-dependent RNA polymerase nucleotide addition cycle in picornaviruses. Enzymes 49, 215–233. doi: 10.1016/bs.enz.2021.06.002 34696833

[B43] Gong L. Chen Y. Hu Z. Hu M. (2013). Testing insecticidal activity of novel chemically synthesized siRNA against *Plutella xylostella* under laboratory and field conditions. PloS One 8, e62990. doi: 10.1371/journal.pone.0062990 23667556 PMC3646892

[B44] Gong C. Yang Z. Hu Y. Wu Q. Wang S. Guo Z.. (2022). Silencing of the *BtTPS* genes by transgenic plant-mediated RNAi to control *Bemisia tabaci* MED. Pest Manag. Sci. 78, 1128–1137. doi: 10.1002/ps.6727 34796637

[B45] Guang S. Bochner A. F. Pavelec D. M. Burkhart K. B. Harding S. Lachowiec J.. (2008). An argonaute transports siRNAs from the cytoplasm to the nucleus. Science. 321, 537–541. doi: 10.1126/science.1157647 18653886 PMC2771369

[B46] Gurunathan S. Kang M. H. Qasim M. Khan K. Kim J. H. (2021). Biogenesis, membrane trafficking, functions, and next generation nanotherapeutics medicine of extracellular vesicles. Int. J. Nanomed. 16, 3357–3383. doi: 10.2147/IJN.S310357 PMC8140893 34040369

[B47] Hamilton A. J. Baulcombe D. C. (1999). A species of small antisense RNA in posttranscriptional gene silencing in plants. Science 286, 950–952. doi: 10.1126/science.286.5441.950 10542148

[B48] Hamilton A. Voinnet O. Chappell L. Baulcombe D. (2015). Two classes of short interfering RNA in RNA silencing. EMBO J. 34, 2590. doi: 10.15252/embj.201570050 26291654 PMC4609188

[B49] Han X. Huang L. J. Feng D. Jiang W. Miu W. Li N. (2019). Plasmodesmata-related structural and functional proteins: the long sought-after secrets of a cytoplasmic channel in plant cell walls. Int. J. Mol. Sci. 20, 2946. doi: 10.3390/ijms20122946 31212892 PMC6627144

[B50] Head G. P. Carroll M. W. Evans S. P. Rule D. M. Willse A. R. Clark T. L.. (2017). Evaluation of smartstax and smartstax PRO maize against western corn rootworm and northern corn rootworm: efficacy and resistance management. Pest Manag. Sci. 73, 1883–1899. doi: 10.1002/ps.4554 28195683

[B51] Henderson I. R. Zhang X. Lu C. Johnson L. Meyers B. C. Green P. J. (2006). Dissecting *Arabidopsis thaliana* DICER function in small RNA processing, gene silencing, and DNA methylation patterning. Nat. Gen. 38, 721–725. doi: 10.1038/ng1804 16699516

[B52] Hines P. A. Gonzalez-Quevedo R. Lambert A. I. O. M. Janssens R. Freischem B. Torren-Edo J.. (2020). Regulatory science to 2025: an analysis of stakeholder responses to the European medicines agency’s strategy. Front. Med. (Lausanne) 7. doi: 10.3389/fmed.2020.00508 PMC7540226 33072771

[B53] Hokaiwado N. Takeshita F. Banas A. Ochiya T. (2008). RNAi-based drug discovery and its application to therapeutics. IDrugs 11, 274–278. 18379962

[B54] Huvenne H. Smagghe G. (2010). Mechanisms of dsRNA uptake in insects and potential of RNAi for pest control: A review. J. Insect Physiol. 56, 227–235. doi: 10.1016/j.jinsphys.2009.10.004 19837076

[B55] Ijaz M. Khan F. Ahmed T. Noman M. Zulfiqar F. Rizwan M.. (2023). Nanobiotechnology to advance stress resilience in plants: current opportunities and challenges. Mater. Today Bio. 22, 100759. doi: 10.1016/j.mtbio.2023.100759 PMC10433128 37600356

[B56] Islam M. T. Davis Z. Chen L. Englaender J. Zomorodi S. Frank J.. (2021). Minicell-based fungal RNAi delivery for sustainable crop protection. Microb. Biotechnol. 14, 1847–1856. doi: 10.1111/1751-7915.13699 33624940 PMC8313293

[B57] Islam M. T. Sherif S. M. (2020). RNAi-based biofungicides as a promising next-generation strategy for controlling devastating gray mold diseases. Int. J. Mol. Sci. 21, 2072. doi: 10.3390/ijms21062072 32197315 PMC7139463

[B58] Ivashuta S. Zhang Y. Wiggins B. E. Seshadri P. R. Segers G. C. Johnson S.. (2015). Environmental RNAi in herbivorous insects. RNA. 21, 840–850. doi: 10.1261/rna.048116.114 25802407 PMC4408792

[B59] Jain R. G. Fletcher S. J. Manzie N. Robinson K. E. Li P. Lu E.. (2022). Foliar application of clay-delivered RNA interference for whitefly control. Nat. Plants 8, 535–548. doi: 10.1038/s41477-022-01152-8 35577960

[B60] James C. (2014). Global Status of Commercialized Biotech/GM Crops: 2014. ISAAA Brief No. 49 (Ithaca, NY: ISAAA).

[B61] Jay S. Petrick-Brent B. T. Aimee L. Jackson-Larry D. K. (2013). Safety assessment of food and feed from biotechnology-derived crops employing RNA-mediated gene regulation to achieve desired traits: A scientific review. Regul. Toxicol. Pharmacol. 2, 167–176. doi: 10.1016/j.yrtph.2013.03.008 23557984

[B62] Jha U. C. Nayyar H. Chattopadhyay A. Beena R. Lone A. A. Naik Y. D.. (2023). Major viral diseases in grain legumes: designing disease resistant legumes from plant breeding and OMICS integration. Front. Plant Sci. 14. doi: 10.3389/fpls.2023.1183505 PMC10204772 37229109

[B63] Joga M. R. Zotti M. J. Smagghe G. Christiaens O. (2016). RNAi efficiency, systemic properties, and novel delivery methods for pest insect control: what we know so far. Front. Physiol. 7. doi: 10.3389/fphys.2016.00553 PMC5112363 27909411

[B64] Jothi K. B. Ramaswamy A. Lincy K. B. Sowbiya M. Muthu-Arjuna S. P. (2023). Recent trends and advances of RNA interference (RNAi) to improve agricultural crops and enhance their resilience to biotic and abiotic stresses. Plant Physiol. Biochem. 194, 600–618. doi: 10.1016/j.plaphy.2022.11.035 36529010

[B65] Kadoić-Balaško M. Mikac K. M. Bažok R. Lemic D. (2020). Modern techniques in Colorado potato beetle (*leptinotarsa decemlineata* say) control and resistance management: history review and future perspectives. Insects 11, 581. doi: 10.3390/insects11090581 32882790 PMC7563253

[B66] Khajuria C. Ivashuta S. Wiggins E. Flagel L. Moar W. Pleau M.. (2018). Development and characterization of the first dsRNA-resistant insect population from western corn rootworm, *Diabrotica virgifera virgifera* LeConte. PloS One 13, e0197059. doi: 10.1371/journal.pone.0197059 29758046 PMC5951553

[B67] Kaur R. Bhunia R. K. Rajam M. V. (2020). MicroRNAs as potential targets for improving rice yield via plant architecture modulation: Recent studies and future perspectives. J. Biosci. 45, 116. doi: 10.1007/s12038-020-00084-9 33051410

[B68] Kedde M. Strasser M. J. Boldajipour B. Oude-Vrielink J. A. Slanchev K. le-Sage C.. (2007). RNA-binding protein Dnd1 inhibits microRNA access to target mRNA. Cell 131, 1273–1286. doi: 10.1016/j.cell.2007.11.034 18155131

[B69] Ketting R. F. (2011). The many faces of RNAi. Dev. Cell. 20, 148–161. doi: 10.1016/j.devcel.2011.01.012 21316584

[B70] Khalid A. Zhang Q. Yasir M. Li F. (2017). Small RNA based genetic engineering for plant viral resistance: application in crop protection. Front. Microbiol. 8. doi: 10.3389/fmicb.2017.00043 PMC5253543 28167936

[B71] Komarova T. Ilina I. Taliansky M. Ershova N. (2023). Nanoplatforms for the delivery of nucleic acids into plant cells. Int. J. Mol. Sci. 24, 16665. doi: 10.3390/ijms242316665 38068987 PMC10706211

[B72] Kong L. Shi X. Chen D. Yang N. Yin C. Yang J.. (2022). Host-induced silencing of a nematode chitin synthase gene enhances resistance of soybeans to both pathogenic *Heterodera glycines* and *Fusarium oxysporum* . Plant Biotechnol. J. 20, 809–811. doi: 10.1111/pbi.13808 35301818 PMC9055809

[B73] Kumar K. Gambhir G. Dass A. Tripathi A. K. Singh A. Jha A. K.. (2020). Genetically modified crops: current status and future prospects. Planta 251, 91. doi: 10.1007/s00425-020-03372-8 32236850

[B74] Kupferschmidt K. (2013). A lethal dose of RNA. Science 341, 732–733. doi: 10.1126/science.341.6147.732 23950525

[B75] Leahy J. Mendelsohn M. Kough J. Jones R. Berckes N. (2014). “Biopesticide oversight and registration of the U.S. Environmental Protection Agency,” in Biopesticides: State of Art and Future Opportunities. Eds. Gross A. Coats J. R. Duke S. O. Seiber J. N. (ACS Publications, Washington, DC), 1–16. doi: 10.1021/bk-2014-1172.ch001

[B76] Liu L. Chen X. (2018). Intercellular and systemic trafficking of RNAs in plants. Nat. Plants 4, 869–878. doi: 10.1038/s41477-018-0288-5 30390090 PMC7155933

[B77] Liu S. Geng S. Li A. Mao Y. Mao L. (2021). RNAi technology for plant protection and its application in wheat. aBIOTECH 2, 365–374. doi: 10.1007/s42994-021-00036-3 36304420 PMC9590511

[B78] Liu G. Kang G. Wang S. Huang Y. Cai Q. (2021). Extracellular vesicles: emerging players in plant defense against pathogens. Front. Plant Sci. 12. doi: 10.3389/fpls.2021.757925 PMC8515046 34659325

[B79] Liu J. Swevers L. Iatrou K. Huvenne H. Smagghe G. (2012). Bombyx mori DNA/RNA non-specific nuclease: expression of isoforms in insect culture cells, subcellular localization and functional assays. J. Insect Physiol. 58, 1166–1176. doi: 10.1016/j.jinsphys.2012.05.016 22709524

[B80] Lundgren J. G. Duan J. J. (2013). RNAi-based insecticidal crops: potential effects on nontarget species. BioScience 63, 657–665. doi: 10.1525/bio.2013.63.8.8

[B81] Ma Z. Z. Zhang Y. H. Li M. S. Chao Z. J. Du X. G. Yan S.. (2023). A first greenhouse application of bacteria-expressed and nanocarrier-delivered RNA pesticide for *Myzus persicae* control. J. Pest Sci. 96, 181–193. doi: 10.1007/s10340-022-01485-5

[B82] Ma Y. F. Zhao Y. Q. Zhou Y. Y. Feng H. Y. Gong L. L. Zhang M. Q.. (2024). Nanoparticle-delivered RNAi-based pesticide target screening for the rice pest white-backed planthopper and risk assessment for a natural predator. Sci. Total Environ. 926, 171286. doi: 10.1016/j.scitotenv.2024.171286 38428617

[B83] Mamta B. Rajam M. V. (2018). RNA Interference: A Promising Approach for Crop Improvement. Biotechnologies of Crop Improvement. (Cham: Springer International Publishing AG) 2, 41–65. doi: 10.1007/978-3-319-90650-8_3

[B84] Mamta Reddy K. R. Rajam M. V. (2016). Targeting chitinase gene of Helicoverpa armigera by host-induced RNA interference confers insect resistance in tobacco and tomato. Plant Mol. Biol. 90, 281–292. doi: 10.1007/s11103-015-0414-y 26659592

[B85] Mamta B. Rajam M. V. (2017). RNAi technology: a new platform for crop pest control. Physiol. Mol. Biol. Plants. 23, 487–501. doi: 10.1007/s12298-017-0443-x 28878489 PMC5567704

[B86] Margis R. Fusaro A. F. Smith N. A. Curtin S. J. Watson J. M. Finnegan E. J.. (2006). The evolution and diversification of Dicers in plants. FEBS Lett. 580, 2442–2450. doi: 10.1016/j.febslet.2006.03.072 16638569

[B87] Mathieu M. Martin-Jaular L. Lavieu G. Théry C. (2019). Specificities of secretion and uptake of exosomes and other extracellular vesicles for cell-to-cell communication. Nat. Cell Biol. 21, 9–17. doi: 10.1038/s41556-018-0250-9 30602770

[B88] Mat-Jalaluddin N. S. Asem M. Harikrishna J. A. Ahmad-Fuaad A. A. H. (2023). Recent progress on nanocarriers for topical-mediated RNAi strategies for crop protection-a review. Molecules 28, 2700. doi: 10.3390/molecules28062700 36985671 PMC10054734

[B89] Matranga C. Zamore P. D. (2007). Small silencing RNAs. Curr. Biol. 17, 789–793. doi: 10.1016/j.cub.2007.07.014 17878043

[B90] McLaughlin M. S. Roy M. Abbasi P. A. Carisse O. Yurgel S. N. Ali S. (2023). Why do we need alternative methods for fungal disease management in plants? Plants (Basel) 12, 3822. doi: 10.3390/plants12223822 38005718 PMC10675458

[B91] Mehlhorn S. Hunnekuhl V. S. Geibel S. (2021). Establishing RNAi for basic research and pest control and identification of the most efficient target genes for pest control: a brief guide. Front. Zool. 18, 60. doi: 10.1186/s12983-021-00444-7 34863212 PMC8643023

[B92] Meister G. Tuschl T. (2004). Mechanisms of gene silencing by double-stranded RNA. Nature 431, 343–349. doi: 10.1038/nature02873 15372041

[B93] Mello C. C. Conte D. Jr. (2004). Revealing the world of RNA interference. Nature 431, 338–342. doi: 10.1038/nature02872 15372040

[B94] Mitter N. Worrall E. A. Robinson K. E. Xu Z. P. Carroll B. J. (2017). Induction of virus resistance by exogenous application of double-stranded RNA. Curr. Opin. Virol. 26, 49–55. doi: 10.1016/j.coviro.2017.07.009 28778033

[B95] Mohr S. E. Perrimon N. (2012). RNAi screening: new approaches, understandings, and organisms. Wiley Interdiscip. Rev. RNA 3, 145–158. doi: 10.1002/wrna.110 21953743 PMC3249004

[B96] Mordukhovich G. Bahar O. (2017). Isolation of outer membrane vesicles from phytopathogenic *Xanthomonas campestris* pv. *campestris* . Bio Protoc. 7, e2160. doi: 10.21769/BioProtoc.2160 PMC8376552 34458473

[B97] Moreno J. A. Hamza E. Guerrero-Hue M. Rayego-Mateos S. García-Caballero C. Vallejo-Mudarra M.. (2021). Non-Coding RNAs in kidney diseases: The long and short of them. Int. J. Mol. Sci. 22, 6077. doi: 10.3390/ijms22116077 34199920 PMC8200121

[B98] Morozov S. Y. Solovyev A. G. Kalinina N. O. Taliansky M. E. (2019). Double-stranded RNAs in plant protection against pathogenic organisms and viruses in agriculture. Acta Naturae 11, 13–21. doi: 10.32607/20758251-2019-11-4-13-21 31993231 PMC6977960

[B99] Mwaka H. S. Bauters L. Namaganda J. Marcou S. Bwesigye P. N. Kubiriba J.. (2023). Transgenic East African highland banana plants are protected against *Radopholus similis* through Host-delivered RNAi. Int. J. Mol. Sci. 24, 12126. doi: 10.3390/ijms241512126 37569502 PMC10418933

[B100] Niño-Sánchez J. Sambasivam P. T. Sawyer A. Hamby R. Chen A. Czislowski E.. (2022). BioClay™ prolongs RNA interference-mediated crop protection against *Botrytis cinerea* . J. Integr. Plant Biol. 64, 2187–2198. doi: 10.1111/jipb.13353 36040241 PMC10464624

[B101] Nitnavare R. B. Bhattacharya J. Singh S. Kour A. Hawkesford M. J. Arora N. (2021). Next generation dsRNA-based insect control: success so far and challenges. Front. Plant Sci. 12. doi: 10.3389/fpls.2021.673576 PMC8558349 34733295

[B102] Numata K. Ohtani M. Yoshizumi T. Demura T. Kodama Y. (2014). Local gene silencing in plants via synthetic dsRNA and carrier peptide. Plant Biotechnol. J. 12, 1027–1034. doi: 10.1111/pbi.12208 24905384

[B103] OECD (2020). Considerations for the environmental risk assessment of the application of sprayed or externally applied dsRNA-based pesticides. Series on Pesticides and Biocides (Paris: Paris). No. 104, ENV/JM/MONO 26. doi: 10.1787/576d9ebb-en

[B104] OECD (2023). Considerations for the human health risk assessment of externally applied dsRNA-based pesticides. (Paris: OECD), ENV/CBC/MONO 2023. doi: 10.1787/54852048-en

[B105] Omolehin O. Raruang Y. Hu D. Han Z. Q. Wei Q. Wang K.. (2021). Resistance to aflatoxin accumulation in maize mediated by host-induced silencing of the aspergillus flavus alkaline protease (alk) gene. J. Fungi (Basel) 7, 904. doi: 10.3390/jof7110904 34829193 PMC8622731

[B106] Ortega-Rivera O. A. Shukla S. Shin M. D. Chen A. Beiss V. Moreno-Gonzalez M. A.. (2021). Cowpea mosaic virus nanoparticle vaccine candidates displaying peptide epitopes can neutralize the severe acute respiratory syndrome coronavirus. ACS Infect. Dis. 7, 3096–3110. doi: 10.1021/acsinfecdis.1c00410 34672530

[B107] Paces J. Nic M. Novotny T. Svoboda P. (2017). Literature review of baseline information to support the risk assessment of RNAi-based GM plants. EFSA Support 14, EN–1246. doi: 10.2903/sp.efsa.2017.EN-1246

[B108] Papadopoulou N. Devos Y. Álvarez-Alfageme F. Lanzoni A. Waigmann E. (2020). Risk assessment considerations for genetically modified RNAi plants: EFSA’s activities and perspective. Front. Plant Sci. 11. doi: 10.3389/fpls.2020.00445 PMC7186845 32373145

[B109] Parker J. S. Barford D. (2006). Argonaute: A scaffold for the function of short regulatory RNAs. Trends Biochem. Sci. 31, 622–630. doi: 10.1016/j.tibs.2006.09.010 17029813

[B110] Parker K. M. Barragán-Borrero V. van-Leeuwen D. M. Lever M. A. Mateescu B. Sander M. (2019). Environmental fate of RNA interference pesticides: adsorption and degradation of double-stranded RNA molecules in agricultural soils. Environ. Sci. Technol. 53, 3027–3036. doi: 10.1021/acs.est.8b05576 30681839

[B111] Parker J. Roe S. Barford D. (2005). Structural insights into mRNA recognition from a PIWI domain-siRNA guide complex. Nature 434, 663–666. doi: 10.1038/nature03462 15800628 PMC2938470

[B112] Pathak V. M. Verma V. K. Rawat B. S. Kaur. B. Babu N. Sharma A.. (2022). Current status of pesticide effects on environment, human health and it’s eco-friendly management as bioremediation: A comprehensive review. Front. Microbiol. 13. doi: 10.3389/fmicb.2022.962619 PMC9428564 36060785

[B113] Preall J. B. Sontheimer E. J. (2005). RNAi: RISC gets loaded. Cell 123, 543–545. doi: 10.1016/j.cell.2005.11.006 16286001

[B114] Qaim M. (2020). Role of new plant breeding technologies for food security and sustainable agricultural development. Appl. Econ. Perspect. Policy 42, 129–150. doi: 10.1002/aepp.13044

[B115] Qiao L. Lan C. Capriotti L. Ah-Fong A. Nino-Sanchez J. Hamby R.. (2021). Spray-induced gene silencing for disease control is dependent on the efficiency of pathogen RNA uptake. Plant Biotechnol. J. 19, 1756–1768. doi: 10.1111/pbi.13589 33774895 PMC8428832

[B116] Qiao W. Medina V. Kuo Y. W. Falk B. W. (2018). A distinct, non-virion plant virus movement protein encoded by a crinivirus essential for systemic infection. mBio 9, e02230-18. doi: 10.1128/mBio.02230-18 30459200 PMC6247084

[B117] Qiao L. Niño-Sánchez J. Hamby R. Capriotti L. Chen A. Mezzetti B.. (2023). Artificial nanovesicles for dsRNA delivery in spray-induced gene silencing for crop protection. Plant Biotechnol. J. 21, 854–865. doi: 10.1111/pbi.14001 36601704 PMC10037145

[B118] Rajam M. V. (2020). RNA silencing technology: A boon for crop improvement. J. Biosci. 45, 118. doi: 10.1007/s12038-020-00082-x 33051412

[B119] Ray P. Sahu D. Aminedi R. Chandran D. (2022). Concepts and considerations for enhancing RNAi efficiency in phytopathogenic fungi for RNAi-based crop protection using nanocarrier-mediated dsRNA delivery systems. Front. Fungal Biol. 3. doi: 10.3389/ffunb.2022.977502 PMC10512274 37746174

[B120] Rodrigues T. B. Mishra S. K. Sridharan K. Barnes E. R. Alyokhin A. Tuttle R.. (2021). First sprayable double-stranded RNA-based biopesticide product targets proteasome subunit beta type-5 in colorado potato beetle (*Leptinotarsa decemlineata*). Front. Plant Sci. 18. doi: 10.3389/fpls.2021.728652 PMC8650841 34887882

[B121] Rodrigues T. B. Petrick J. S. (2020). Safety considerations for humans and other vertebrates regarding agricultural uses of externally applied RNA molecules. Front. Plant Sci. 11. doi: 10.3389/fpls.2020.00407 PMC7191066 32391029

[B122] Rodriguez Coy L. Plummer K. M. Khalifa M. E. MacDiarmid R. M. (2022). Mycovirus-encoded suppressors of RNA silencing: Possible allies or enemies in the use of RNAi to control fungal disease in crops. Front. Fungal Biol. 3. doi: 10.3389/ffunb.2022.965781 PMC10512228 37746227

[B123] Romeis J. Widmer F. (2020). Assessing the risks of topically applied dsRNA-based products to non-target arthropods. Front. Plant Sci. 11. doi: 10.3389/fpls.2020.00679 PMC7289159 32582240

[B124] Samad A. F. A. Kamaroddin M. F. Sajad M. (2021). Cross-kingdom regulation by plant microRNAs provides novel insight into gene regulation. Adv. Nutr. 12, 197–211. doi: 10.1093/advances/nmaa095 32862223 PMC7850022

[B125] San-Miguel K. Scott J. G. (2016). The next generation of insecticides: dsRNA is stable as a foliar-applied insecticide. Pest Manag. Sci. 72, 801–809. doi: 10.1002/ps.4056 26097110

[B126] Sarkar A. Roy-Barman S. (2021). Spray-induced silencing of pathogenicity gene MoDES1 via exogenous double-stranded RNA can confer partial resistance against fungal blast in Rice. Front. Plant Sci. 12. doi: 10.3389/fpls.2021.733129 PMC8662628 34899771

[B127] Schwartz S. H. Hendrix B. Hoffer P. Sanders R. A. Zheng W. (2020). Carbon dots for efficient small interfering RNA delivery and gene silencing in plants. Plant Physiol. 184, 647–657. doi: 10.1104/pp.20.00733 32764133 PMC7536711

[B128] Setten R. L. Rossi J. J. Han S. P. (2019). The current state and future directions of RNAi-based therapeutics. Nat. Rev. Drug Discovery 18, 421–446. doi: 10.1038/s41573-019-0017-4 30846871

[B129] Sikandar A. Khanum T. A. Wang Y. (2021). Biodiversity and community analysis of plant-parasitic and free-living nematodes associated with maize and other rotational crops from Punjab, Pakistan. Life (Basel) 11, 1426. doi: 10.3390/life11121426 34947957 PMC8706526

[B130] Song X. S. Gu K. X. Duan X. X. Xiao X. M. Hou Y. P. Duan Y. B.. (2018). A myosin5 dsRNA that reduces the fungicide resistance and pathogenicity of *Fusarium asiaticum* . Pestic. Biochem. Physiol. 150, 1–9. doi: 10.1016/j.pestbp.2018.07.004 30195381

[B131] Spit J. Philips A. Wynant N. Santos D. Plaetinck G. Vanden Broeck J. (2017). Knockdown of nuclease activity in the gut enhances RNAi efficiency in the Colorado potato beetle, *Leptinotarsa decemlineata*, but not in the desert locust, *Schistocerca gregaria* . Insect Biochem. Mol. Biol. 81, 103–116. doi: 10.1016/j.ibmb.2017.01.004 28093313

[B132] Statello L. Guo C. J. Chen L. L. Huarte M. (2020). Gene regulation by long non-coding RNAs and its biological functions. Nat. Rev. Mol. Cell Biol. 22, 96–118. doi: 10.1038/s41580-020-00315-9 33353982 PMC7754182

[B133] Subha D. AnuKiruthika R. Sreeraj H. Tamilselvi K. S. (2023). Plant exosomes: nano conveyors of pathogen resistance. Discovery Nano. 18, 146. doi: 10.1186/s11671-023-03931-4 PMC10689327 38032422

[B134] Talap J. Zhao J. Shen M. Song Z. Zhou H. Kang Y.. (2021). Recent advances in therapeutic nucleic acids and their analytical methods. J. Pharm. BioMed. Anal. 206, 114368. doi: 10.1016/j.jpba.2021.114368 34571322

[B135] Tatematsu M. Funami K. Seya T. Matsumoto M. (2018). Extracellular RNA sensing by pattern recognition receptors. J. Innate Immun. 10, 398–406. doi: 10.1159/000494034 30404092 PMC6784046

[B136] Tayler A. Heschuk D. Giesbrecht D. Park J. Y. Whyard S. (2019). Efficiency of RNA interference is improved by knockdown of dsRNA nucleases in tephritid fruit flies. Open Biol. 9, 190198. doi: 10.1098/rsob.190198 31795920 PMC6936256

[B137] Tenllado F. Díaz-Ruíz J. R. (2001). Double-stranded RNA-mediated interference with plant virus infection. J. Virol. 75, 12288–12297. doi: 10.1128/JVI.75.24.12288-12297.2001 11711619 PMC116125

[B138] Timani K. Bastarache P. Morin P. J. (2023). Leveraging RNA interference to impact insecticide resistance in the Colorado potato beetle, *Leptinotarsa decemlineata* . Insects 14, 418. doi: 10.3390/insects14050418 37233046 PMC10231074

[B139] Tran T. M. Chng C. P. Pu X. Ma Z. Han X. Liu X.. (2022). Potentiation of plant defense by bacterial outer membrane vesicles is mediated by membrane nanodomains. Plant Cell. 34, 395–417. doi: 10.1093/plcell/koab276 34791473 PMC8846181

[B140] Tyagi S. Sharma S. Ganie S. A. Tahir M. Mir R. R. Pandey R. (2019). Plant microRNAs: biogenesis, gene silencing, web-based analysis tools and their use as molecular markers. 3 Biotech. 9, 413. doi: 10.1007/s13205-019-1942-y PMC6811466 31696018

[B141] Uslu V. V. Bassler A. Krczal G. Wassenegger M. (2020). High-pressure-sprayed double stranded RNA does not induce RNA interference of a reporter gene. Front. Plant Sci. 11. doi: 10.3389/fpls.2020.534391 PMC7773025 33391294

[B142] Vatanparast M. Kim Y. (2017). Optimization of recombinant bacteria expressing dsRNA to enhance insecticidal activity against a lepidopteran insect, *Spodoptera exigua* . PloS One 12, e0183054. doi: 10.1371/journal.pone.0183054 28800614 PMC5553977

[B143] Walawage S. L. Britton M. T. Leslie C. A. Uratsu S. L. Li Y. Dandekar A. M. (2013). Stacking resistance to crown gall and nematodes in walnut rootstocks. BMC Genomics 14, 668. doi: 10.1186/1471-2164-14-668 24083348 PMC3852553

[B144] Wang M. Jin H. (2017). Spray-induced gene silencing: a powerful innovative strategy for crop protection. Trends Microbiol. 25, 4–6. doi: 10.1016/j.tim.2016.11.011 27923542 PMC5182084

[B145] Wang M. Weiberg A. Lin F. M. Thomma B. P. Huang H. D. Jin H. (2016). Bidirectional cross-kingdom RNAi and fungal uptake of external RNAs confer plant protection. Nat. Plants 2, 16151. doi: 10.1038/nplants.2016.151 27643635 PMC5040644

[B146] Wang M. Wu L. Mei Y. Zhao Y. Ma Z. Zhang X.. (2020). Host-induced gene silencing of multiple genes of *Fusarium graminearum* enhances resistance to *Fusarium* head blight in wheat. Plant Biotechnol. J. 18, 2373–2375. doi: 10.1111/pbi.13401 32436275 PMC7680546

[B147] Wang Y. Yan Q. Lan C. Tang T. Wang K. Shen J.. (2023). Nanoparticle carriers enhance RNA stability and uptake efficiency and prolong the protection against *Rhizoctonia solani* . Phytopathol. Res. 5, 2. doi: 10.1186/s42483-023-00157-1

[B148] Wassenegger M. Krczal G. (2006). Nomenclature and functions of RNA-directed RNA polymerases. Trends Plant Sci. 11, 142–151. doi: 10.1016/j.tplants.2006.01.003 16473542

[B149] Waterhouse P. M. Graham M. W. Wang M.-B. (1998). Virus resistance and gene silencing in plants can be induced by simultaneous expression of sense and antisense RNA. Proc. Natl. Acad. Sci. U. S. A. 95, 13959–13964. doi: 10.1073/pnas.95.23.13959 9811908 PMC24986

[B150] Weiberg A. Wang M. Lin F. M. Zhao H. Zhang Z. Kaloshian I.. (2013). Fungal small RNAs suppress plant immunity by hijacking host RNA interference pathways. Science 342, 118–123. doi: 10.1126/science.1239705 24092744 PMC4096153

[B151] Wen H. G. Zhao J. H. Zhang B. S. (2023). Microbe-induced gene silencing boosts crop protection against soil-borne fungal pathogens. Nat. Plants 9, 1409–1418. doi: 10.1038/s41477-023-01507-9 37653339

[B152] Willow J. Soonvald L. Sulg S. Kaasik R. Silva A. I. Taning C. N. T.. (2021). RNAi efficacy is enhanced by chronic dsRNA feeding in pollen beetle. Commun. Biol. 4, 444. doi: 10.1038/s42003-021-01975-9 33824392 PMC8024372

[B153] Wilson R. C. Doudna J. A. (2013). Molecular mechanisms of RNA interference. Annu. Rev. Biophys. 42, 217–239. doi: 10.1146/annurev-biophys-083012-130404 23654304 PMC5895182

[B154] Wingard S. A. (1928). Hosts and symptoms of ring spot, a virus disease of plants. J. Agric. Res. 37, 127–153.

[B155] Wozniak C. A. McClung G. Gagliardi J. Segal M. Matthews K. (2024). “Regulation of genetically engineered microorganisms under FIFRA, FFDCA and TSCA,” in Regulation of Agricultural Biotechnology: The United States and Canada. Eds. Wozniak C. A. McHughen A. (Springer, Heidelberg), 57–94. doi: 10.1007/978-94-007-2156-2_4

[B156] Wu K. Xu C. Li T. Ma H. Gong J. Li X.. (2023). Application of nanotechnology in plant genetic engineering. Int. J. Mol. Sci. 24, 14836. doi: 10.3390/ijms241914836 37834283 PMC10573821

[B157] Wu L. Liu S. Qi H. Cai H. Xu M. (2020). Research progress on plant long non-coding RNA. Plants (Basel). 9, 408. doi: 10.3390/plants9040408 32218186 PMC7237992

[B158] Yan Y. Ham B. K. (2022). The mobile small RNAs: Important messengers for long-distance communication in plants. Front. Plant Sci. 13. doi: 10.3389/fpls.2022.928729 PMC9247610 35783973

[B159] Yan S. Qian J. Cai C. Ma Z. Z. Li J. H. Yin M. Z.. (2020). Spray method application of transdermal dsRNA delivery system for efficient gene silencing and pest control on soybean aphid *Aphis glycines* . J. Pest Sci. 93, 449–459. doi: 10.1007/s10340-019-01157-x

[B160] Yan S. Ren B. Y. Shen J. (2021). Nanoparticle-mediated double-stranded RNA delivery system: A promising approach for sustainable pest management. Insect Sci. 28, 21–34. doi: 10.1111/1744-7917.12822 32478473

[B161] Yang J. Hwang I. Lee E. Shin S. J. Lee E. J. Rhee J. H.. (2020). Bacterial outer membrane vesicle-mediated cytosolic delivery of flagellin triggers host NLRC4 canonical inflammasome signaling. Front. Immunol. 11. doi: 10.3389/fimmu.2020.581165 PMC7708323 33312172

[B162] Yong J. X. Zhang R. Bi S. N. Li P. Sun L. Y. Mitter N.. (2021). Sheet-like clay nanoparticles deliver RNA into developing pollen to efficiently silence a target gene. Plant Physiol. 2, 886–899. doi: 10.1093/plphys/kiab303 PMC8491087 34608968

[B163] Yoshida K. Suehiro Y. Dejima K. Yoshina S. Mitani S. (2023). Distinct pathways for export of silencing RNA in *Caenorhabditis elegans* systemic RNAi. iScience 26, 108067. doi: 10.1016/j.isci.2023.108067 37854694 PMC10579535

[B164] Yu Y. Jia T. Chen X. (2017). The ‘how’ and ‘where’ of plant microRNAs. New Phytol. 216, 1002–1017. doi: 10.1111/nph.14834 29048752 PMC6040672

[B165] Zhang H. Goh N. S. Wang J. W. Pinals R. L. González-Grandío E. Demirer G. S.. (2022). Nanoparticle cellular internalization is not required for RNA delivery to mature plant leaves. Nat. Nanotechnol. 17, 197–205. doi: 10.1038/s41565-021-01018-8 34811553 PMC10519342

[B166] Zotti M. J. Smagghe G. (2015). RNAi technology for insect management and protection of beneficial insects from diseases: lessons, challenges and risk assessments. Neotrop. Entomol. 44, 197–213. doi: 10.1007/s13744-015-0291-8 26013264

